# Genomic studies with preselected markers reveal dominance effects influencing growth traits in *Eucalyptus nitens*

**DOI:** 10.1093/g3journal/jkab363

**Published:** 2021-11-09

**Authors:** Bala R Thumma, Kelsey R Joyce, Andrew Jacobs

**Affiliations:** 1 Gondwana Genomics Pty Ltd, Canberra, ACT 2600, Australia; 2 Forico Pty Ltd, Kings Meadows, TAS 7249, Australia

**Keywords:** inbreeding depression, genomic selection, identity-by-descent, identity-by-state, ssGBLUP, GBLUP

## Abstract

Genomic selection (GS) is being increasingly adopted by the tree breeding community. Most of the GS studies in trees are focused on estimating additive genetic effects. Exploiting the dominance effects offers additional opportunities to improve genetic gain. To detect dominance effects, trait-relevant markers may be important compared to nonselected markers. Here, we used preselected markers to study the dominance effects in a *Eucalyptus nitens* (*E. nitens*) breeding population consisting of open-pollinated (OP) and controlled-pollinated (CP) families. We used 8221 trees from six progeny trials in this study. Of these, 868 progeny and 255 parents were genotyped with the *E. nitens* marker panel. Three traits; diameter at breast height (DBH), wood basic density (DEN), and kraft pulp yield (KPY) were analyzed. Two types of genomic relationship matrices based on identity-by-state (IBS) and identity-by-descent (IBD) were tested. Performance of the genomic best linear unbiased prediction (GBLUP) models with IBS and IBD matrices were compared with pedigree-based additive best linear unbiased prediction (ABLUP) models with and without the pedigree reconstruction. Similarly, the performance of the single-step GBLUP (ssGBLUP) with IBS and IBD matrices were compared with ABLUP models using all 8221 trees. Significant dominance effects were observed with the GBLUP-AD model for DBH. The predictive ability of DBH is higher with the GBLUP-AD model compared to other models. Similarly, the prediction accuracy of genotypic values is higher with GBLUP-AD compared to the GBLUP-A model. Among the two GBLUP models (IBS and IBD), no differences were observed in predictive abilities and prediction accuracies. While the estimates of predictive ability with additive effects were similar among all four models, prediction accuracies of ABLUP were lower than the GBLUP models. The prediction accuracy of ssGBLUP-IBD is higher than the other three models while the theoretical accuracy of ssGBLUP-IBS is consistently higher than the other three models across all three groups tested (parents, genotyped, and nongenotyped). Significant inbreeding depression was observed for DBH and KPY. While there is a linear relationship between inbreeding and DBH, the relationship between inbreeding and KPY is nonlinear and quadratic. These results indicate that the inbreeding depression of DBH is mainly due to directional dominance while in KPY it may be due to epistasis. Inbreeding depression may be the main source of the observed dominance effects in DBH. The significant dominance effect observed for DBH may be used to select complementary parents to improve the genetic merit of the progeny in *E. nitens*.

## Introduction

*Eucalyptus nitens* is a temperate plantation tree species grown predominantly in Southern Australia and Chile. *E. nitens* is the second most widely grown plantation hardwood species in Australia (ABERES 2018). *E. nitens* is primarily grown to produce pulpwood but it is increasingly being utilized for solid wood production. While several studies have analyzed genetic parameters of growth and wood (Borralho *et al.* 1997; Kube *et al.* 2002; Hamilton and Potts 2008; Volker *et al.* 2008), a few studies have used genomics to study genetic parameters and prediction accuracies in *E. nitens* (Klápště *et al.* 2017, 2018, 2020; Suontama *et al.* 2019).

Genomic selection (GS) has become routine in animal breeding, and it is increasingly being adopted by the tree breeding community (Beaulieu *et al.* 2014; Isik *et al.* 2016; Grattapaglia *et al.* 2018; Thavamanikumar *et al.* 2020). Due to long generational intervals in trees, GS is anticipated to have a large impact on tree breeding. In addition to aiding in quality control in breeding programs, such as correcting for pedigree and clonal errors, the main benefits of GS in tree breeding include increased accuracy of the estimated molecular breeding values (MBVs), reduction of breeding cycle through early selection and increased selection intensity which will all lead to significant improvements in genetic gain (Grattapaglia *et al.* 2018).

One of the methods used for the application of GS in tree breeding is the genomic best linear unbiased prediction (GBLUP) model. In GBLUP, the pedigree-based numerator relationship matrix (NRM) is replaced by the genomic relationship matrix (GRM) for estimating breeding values. MBVs estimated with the GBLUP will be more accurate as the GRM captures the realized relationships between the individuals within a family and relationships among individuals from different families leading to increased accuracy of the estimated MBVs (VanRaden 2008; Goddard and Hayes 2009; Hayes *et al.* 2009). In most of the GS studies in forest trees, GBLUP was mainly used to estimate additive effects (Resende *et al.* 2012; Müller *et al.* 2017; Suontama *et al.* 2019). However, dominance plays a significant role in the genetic control of growth traits compared to wood traits. Several studies in forest trees used GBLUP to estimate dominance effects (Zapata-Valenzuela *et al.* 2013; de Almeida Filho *et al.* 2016, 2019; Klápště *et al.* 2017). Muñoz *et al.* (2014) have reported that the inclusion of nonadditive effects in the genomic model improved the precision of genetic parameters of height in *Pinus taeda*. However, Lenz *et al.* (2020) did not find significant dominance effects using polycross and full-sib families with random markers. Dominance effects, when present, can be utilized through the cloning of target trees. Dominance can also be used for mate allocation. However, in most of the studies where significant dominance effects are observed, the inclusion of them in the prediction models did not result in the significant improvement of the prediction accuracies (Bouvet *et al.* 2016; Resende *et al.* 2017; Gamal El-Dien *et al.* 2018; Tan *et al.* 2018). Accuracies of the models with the dominance effects will only be higher than the additive models when the estimated dominance effects are high (de Almeida Filho *et al.* 2016). In a recent study (Thavamanikumar *et al.* 2020), we have identified significant dominance effects for growth traits in *Eucalyptus pellita* (*E. pellita*) using preselected markers. Higher estimates of predictive ability were observed for growth traits when dominance effects were included in the prediction model.

Single-step genomic best linear unbiased prediction (ssGBLUP) models are increasingly being tested in forest trees (Cappa *et al.* 2017, 2019; Ratcliffe *et al.* 2017; Klápště *et al.* 2018; Callister *et al.* 2021). In ssGBLUP, the GRM of the genotyped samples is combined with the NRM of the nongenotyped samples to generate *H*-matrix for estimating breeding values. Thus, in ssGBLUP, information gathered from all the individuals of the entire breeding population is effectively used in the analysis (Legarra *et al.* 2009). In contrast, estimation of the breeding values is restricted only to the genotyped samples in GBLUP. In ssGBLUP, genomic relationships of genotyped samples are projected onto nongenotyped samples leading to higher accuracy of the breeding values estimated with ssGBLUP compared to ABLUP. Several studies have indicated higher accuracies with ssGBLUP (Aguilar *et al.* 2010; Chen *et al.* 2011; Vitezica *et al.* 2011; Christensen *et al.* 2012). In tree breeding, ssGBLUP can be used to estimate breeding values of parents in environments where they were not progeny tested. Recently, Callister *et al.* (2021) were able to establish the links between the two distinct breeding programs of *Eucalyptus globulus* (*E. globulus*) with the combined *H*-matrix. For this, they used the genotype data of a small number of parents from both the programs to develop a combined *H*-matrix. By using the *H*-matrix in ssGBLUP they were able to estimate breeding values for parents in environments where they were not progeny tested. However, the application of ssGBLUP in tree breeding needs to be approached cautiously. In animal breeding where ssGBLUP is being implemented, the pedigrees are deep and accurate pedigree records are generally maintained. In contrast, most of the tree breeding populations are shallow (Gamal El-Dien *et al.* 2016) and pedigree errors are common (Muñoz *et al.* 2014). This will limit the application of ssGBLUP in tree breeding as pedigree going back several generations with well-connected families and pedigree without errors are important for implementing ssGBLUP. Unrecognized or hidden relationships and pedigree errors among the nongenotyped individuals in ssGBLUP will lead to low accuracy of the predicted breeding values (Klápště *et al.* 2018).

In most of the studies using GBLUP or ssGBLUP models, a GRM based on identity-by-state (IBS) was used. Very few studies have used genomic relationship matrices based on identity-by-descent (IBD) to estimate genetic parameters. Models with an IBD matrix use marker information to track haplotype blocks within the known pedigree using linkage analysis. The accuracy of this method depends on the extent of the family structure. One of the advantages of this method is that it does not require dense markers due to the limited number of recombinations that occur between the generations (Vela-Avitúa *et al.* 2015). Mixed results were observed comparing the prediction accuracies with IBS and IBD relationship measures (Luan *et al.* 2012; Vela-Avitúa *et al.* 2015; Forneris *et al.* 2016). Vela-Avitúa *et al.* (2015) have observed a higher accuracy of GS with the IBS matrix compared to the models with the IBD matrix at a higher marker density. At lower marker densities, however, the accuracy of IBS-GS declined while the accuracy of IBD-GS remained stable. Luan *et al.* (2012) observed similar accuracies between the two models using IBS and IBD matrices. However, Forneris *et al.* (2016) have observed higher accuracies of breeding value predictions with the IBD matrix in ssGBLUP. In a recent study in *Eucalyptus dunnii* (*E. dunnii*), higher breeding value accuracies with lower bias were observed for growth and stem form using the ssGBLUP model with IBD compared to the IBS matrix (Jurcic *et al.* 2021). The authors recommend using ssGBLUP with an IBD matrix in open-pollinated forest tree evaluations.

In a previous project (S. Southerton, S. Thavamanikumar, B. Thumma, unpublished), we developed marker panels for several species of *Eucalyptus* including *E. nitens* with preselected markers. Markers associated with different traits such as growth and wood traits were identified by sequencing samples from extremes of trait distribution. Candidate markers with the potential association with the traits were selected by comparing allele frequency differences that are consistent across different populations. Marker panels were developed with the selected candidate markers and genotyped with the “Targeted genotyping by sequencing (TGS)” method. Thus, the marker panels developed with the preselected markers consist of trait-relevant markers of different traits.

In this study, we used preselected markers associated with various wood and growth traits to compare the predictive performance of the ABLUP, GBLUP, and ssGBLUP models in *E. nitens*. We have tested the ability of the preselected markers to identify the dominance effects of growth and wood traits. We have also compared the prediction accuracies of GBLUP and ssGBLUP models using IBS and IBD relationship matrices.

## Materials and methods

### Population data

A third-generation breeding population consisting of 8221 trees from six progeny trials in Tasmania were used in this study (Table  1). In total, 199 families from 330 parents were used in this study. Of the 199 families, 139 were OP families and 60 were CP families. Of the 60 CP families, 33 were polycrosses generated with pollen pools (polymix). The number of trees per family ranged from 5 to 69 with an average of 41 trees.

**Table 1:** Details of the breeding population used in this study

		Families			Trials					
		No. of trees	OP	CP	Blythe	Hudler	Middlesex	Kelatier	Loudwater	Myrtlebank
Parents	Genotyped	255	184	NA	NA	NA	NA	NA	NA	NA
	Nongenotyped	75	NA	NA	NA	NA	NA	NA	NA	NA
	Total	330	NA	NA	NA	NA	NA	NA	NA	NA
Progeny	Genotyped	868	112	60	201	398	269	NA	NA	NA
	Nongenotyped	7353	139	57	1127	369	333	1506	1981	2037
	Total	8221	251	117	1328	767	602	1506	1981	2037
	Age (year, months)	NA	NA	NA	18.6	8.8	14.9	3.8	8.9	3.9
	Number of replicates	NA	NA	NA	8	6	5	8	8	8
	Number of rows	NA	NA	NA	32	30	14	77	48	46
	Number of columns	NA	NA	NA	104	74	46	58	107	125
	DBH-cm (SD)	NA	NA	NA	20.6 (6.1)	17.8 (3.4)	16.3 (4.6)	11.6 (2.6)	16.4 (4.0)	9.6 (2.5)
	KPY-%(SD)	NA	NA	NA	52.4 (1.1)	53.0 (1.0)	50.2 (0.9)	NA	NA	NA
	DEN-kg/m^3^(SD)	NA	NA	NA	489.4 (19.4)	462.4 (20.5)	493.3 (18.9)	NA	NA	NA

From the total population of 8221 trees, 868 progeny from 172 families were genotyped. In addition, 547 parents of which 255 were the parents of the families used in this study were also genotyped. In total, 1415 trees were genotyped. Trees for genotyping were selected based on the criteria that they all have three traits measured including growth and wood quality traits. Families were selected based on the criteria that they have a minimum of 8 trees per family. The number of trees genotyped per family ranged from 1 to 15 with an average of 5 trees per family.

Diameter at breast height (DBH-cm) was measured across all the 8221 trees used in this study while wood density (DEN-kg/m^3^) and kraft pulp yield (KPY-%) were measured only in 868 genotyped trees. Wood density (DEN) was measured using a Resistograph tool (Downes *et al.* 2018). Wood core samples were used to estimate KPY with Near Infra-Red spectral analysis (Downes *et al.* 2010). DBH data from six trials and wood trait data from three trials were used in this study (Table  1).

### Preselection of markers and genotyping

In a previous project (S. Southerton, S. Thavamanikumar, B. Thumma, unpublished), markers with potential association with different traits of *E. nitens* were identified. For this, field trials consisting of several first-generation families from three contrasting sites were used as a discovery population for the identification of the markers. In total, 400 to 600 trees from each trial were used. The families used in these trials represent a cross-section of genetic material used in *E. nitens* breeding programs in Australia. DNA samples from trait extremes were pooled and subjected to high throughput sequencing. Candidate genes affecting growth and wood traits from earlier studies (Thumma *et al.* 2012; Thavamanikumar *et al.* 2014) were used to capture the gene regions before high throughput sequencing. The three most important traits in forest tree breeding *i.e*., DBH, pulp yield, wood density were used in making the DNA pools. Pools were made separately for each trait. Three biological replicates were made from each pool. Thus, 18 pools per trait were used in high throughput sequencing. Candidate markers showing the potential association with the traits were identified by comparing allele frequencies of pooled samples from trait extremes. The markers showing the highest differences between the pools were selected as the potential candidate markers. To identify the markers that are robust across different environments, markers with consistent effects *i.e*., marker effects in the same direction across all trials from different environments were selected. By analyzing hundreds of thousands of variants, markers showing the largest effect were selected for developing custom marker panels. The selected markers include single nucleotide polymorphisms (SNPs) as well as small biallelic INDELs. Thus, the *E. nitens* marker panel consists of preselected markers associated with growth and wood traits. Marker panels for genotyping were developed with the probes to capture short genomic regions spanning the selected candidate markers. As small genomic regions were sequenced, novel markers within the sequenced regions in addition to the targeted markers will also be genotyped with this method.

TGS was used to genotype the captured genomic regions at Gondwana Genomics Pty Ltd, Canberra. As specific regions were used for sequencing, high coverage (>75x) is generally obtained across the captured regions with this method. DNA from 868 progeny and 547 parents was used for genotyping with the *E. nitens* marker panel. After filtering for markers with <90% call rate and minor allele frequency of <0.01, 3514 markers were selected for downstream analyses. On average each marker had 3% of missing data. Missing marker genotypes were imputed based on average genotype content.

### Pedigree correction and reconstruction

We used the GRM from parental and progeny data to confirm or to identify the pedigree errors. Some of the documented relationships between the parents were corrected by the pedigree analysis with the markers. Pedigree errors among the progeny were corrected by identifying the right parents for some families. Pedigree errors were identified by comparing the genomic relationship coefficients between the members of a family and the documented parents. Individuals showing low relationship coefficients compared to the average relationship coefficients of a family and the documented parents were identified as errors. To identify the paternal parents of polycrosses, genomic relationship coefficients of a family were compared with those of the parents within the pollen mix groups used for generating polycrosses. Genomic relationship coefficients of 0.30–0.5 were used to confirm the parental and progeny relationships.

### Inbreeding depression

Inbreeding was estimated using all the markers. Inbreeding was estimated with the “het” function of the “PLINK” 2.0 package (Purcell *et al.* 2007). To estimate inbreeding depression, linear and quadratic regressions were performed with the phenotype data and the estimates of inbreeding coefficients. To compare the best fit between different models, a partial *F*-test was performed. All these analyses were performed in R.

### Statistical models

Phenotypic data of DBH, DEN, and KPY were adjusted to account for the site and spatial effects. The phenotypic data were adjusted for structured environmental effects estimated from a model that combined design effects (augmented with row and columns within large design features) and spatial effects detected using a separable two-dimensional auto-regressive model (Dutkowski *et al.* 2002). To account for age differences between the trials, trait data were standardized to zero mean and unit variance. Adjusted and standardized phenotypes were used in all subsequent analyses. Four models were used to estimate the genetic parameters and breeding values. Two GBLUP models with IBS and IBD relationship matrices and two ABLUP models based on the documented pedigree, one with pedigree correction (ABLUP-PR) and another without the pedigree correction (ABLUP) were used to estimate the genetic parameters and breeding values in the genotyped trees. In addition, two single-step models (ssGBLUP-IBS and ssGBLUP-IBD) and two ABLUP models (ABLUP-PR with pedigree correction and ABLUP without the pedigree correction) with the DBH of all trees were also used.

The following individual tree mixed model with additive and dominance effects was used to estimate the genetic parameters.

$$y=X\mu \;+Z_{a\;}a+Z_{d}d+\epsilon$$

Where y is the phenotype adjusted for site and spatial effects, μ is the intercept, a is a vector of the random additive and d is a vector of random dominance genetic effects of individual trees, ϵ is the vector of random residual effects.$\;X,\;Z_{a\;}$, and $Z_{d}$ are the incident matrices relating to fixed and random additive and dominance effects, a is distributed as $a\;∼\;N\;(0,A{\sigma^{2}}_{a})$ where ${\sigma^{2}}_{a}$ is the additive genetic variance and A is the additive genetic relationship matrix, d is distributed as $d\;∼\;N\;(0,D{\sigma^{2}}_{d})$ where ${\sigma^{2}}_{d}\;$is the dominance genetic variance and D is the average dominance genetic relationship matrix ϵ is distributed as $\epsilon \;∼\;N\;(0,\;I\;\sigma^{2}\epsilon )$ where I is an identity matrix and $\sigma^{2}\varepsilon$ is the residual variance. In ABLUP, A and D matrices are the average NRM and dominance relationship matrices derived from pedigree; in GBLUP, the A and D matrices are replaced by $G_{A}$ and $G_{D}$ genomic relationship matrices derived from the marker data; in ssGBLUP, the A matrix is replaced by the *H*-matrix.

#### GBLUP:

Two types of genomic relationship matrices, *i.e.*, IBS and IBD were used in GBLUP. The IBS GRM is based on the VanRaden method (VanRaden 2008) and it is calculated as,

GA= WaW'a2∑pj(1-pj)j=1m,

where $W_{a}$ is a matrix of the SNP markers with $W_{\mathrm{aij}}=\{2-2p_{j},1-2p_{j},-2p_{j}\}$, where $W_{\mathrm{aij}}$ represents the elements of $W_{a}$ matrix at ith row and jth column. $p_{j}$ is the allele frequency of jth marker.

The IBD based GRM was obtained with the IBDLD v3.37 software package (Han and Abney 2011). “GIBDLD” option was used to estimate IBD GRM which uses marker information and the genetic map without the pedigree information. IBDLD estimates the IBD relationship matrix by accounting for linkage disequilibrium (LD) between the markers using all markers (Han and Abney 2013). To estimate the LD pattern number of consecutive loci (-ploci n) and distance (-dist k) were used jointly. For this, we have used the default values of *n* = 10 and *k* = 2 cM. The physical position of the markers was obtained by mapping the sequenced regions to the *E. grandis* reference genome. As we do not have the markers mapped on a genetic linkage map, we have converted the physical position into an approximate genetic position by taking 1 Mb = 1 cM.

#### GBLUP-AD

Marker-based dominance matrix was calculated according to Vitezica *et al.* (2013) as,

$$G_{D}=\;\frac{W_{d}{W'}_{d}}{{\sum_{j}\left(2p_{j}q_{j}\right)}^{2}},$$

where $W_{d}$ is a matrix of the SNP markers with $W_{\mathrm{dij}}=\{-2{p_{j}}^{2},2p_{j}q_{j},-2{q_{j}}^{2}\}$, where $W_{\mathrm{dij}}$ represents the elements of $W_{d}$ matrix at ith row and jth column. $p_{j}$ is the allele frequency of jth marker, $q_{j}$ is 1 – $p_{j}$.

#### Combined H-matrix

The combined *H*-matrix for ssGBLUP was obtained with the HIBLUP software package (Yin *et al.* 2019). The IBS GRM ($G_{A}$**)** based on the VanRaden method and the average additive relationship matrix from pedigree (*A*-matrix) were used for developing the *H*-matrix. For *H*-matrix with IBD, the GRM from IBDLD was used. *A*-matrix from an error-corrected pedigree was used to develop the *H*-matrix.

The combined *H*-matrix for ssGBLUP was developed using the following equation.

$$H=\left(\begin{array}{cc} A_{11}-A_{12}A_{22}^{-1}A_{21}+A_{12}A_{22}^{-1}{G_{w}A}_{22}^{-1}A_{21} & A_{12}A_{22}^{-1}G_{w}\\ {G_{w}A}_{22}^{-1}A_{21} & G_{w} \end{array}\right)$$

For this, individuals were assigned to different groups based on available information; the group with the subscript “1” represent individuals that only had pedigree information and the group with the subscript “2” represent individuals that had only genomic information. A_11_ and A_22_ represent relationships among individuals within the group “1” and the group “2,” respectively, A_12_ represents relationships among individuals between the group “1” and “2”, and A_21_ is the transpose of A_12_.

To have the same scale between *G* and A_22_ the following adjustment was made to the *G* matri*x*.

Ga= βG+α

The adjustment factors β and α were derived from the following equation (Christensen *et al.* 2012):

$$\begin{array}{c} \text{Avg}.\text{diag}\left(G\right)\beta +\alpha =\text{Avg}.\text{diag}\left({\text{A}}_{\text{22}}\right)\text{ and}\\ \text{Avg}.\text{offdiag}\left(G\right)\beta +\alpha \;=\text{Avg}.\text{offdiag}\left({\text{A}}_{\text{22}}\right) \end{array}$$

Where: Avg.diag is the average of diagonals and Avg.offdiag is the average of off-diagonal elements.

The rescaled Ga matrix was weighted (Aguilar *et al.* 2010) as follows.

$$Gw=0.95Ga+0.05A_{22}$$

The weighted Gw was used to develop the *H*-matri*x*

The additive relationship matrix and dominance matrix from the pedigree were developed using AGHmatrix software (Amadeu *et al.* 2016). Similarly, additive GRM (IBS) and dominance GRM were also developed with the AGHmatrix package. All the relationship matrices developed with different packages were used in the “SOMMER” package (Covarrubias-Pazaran 2016) to estimate genetic parameters and breeding values. Variance components were estimated using restricted maximum likelihood (REML) with Direct-inversion Newton-Raphson (NR) method.

Narrow-sense heritability (*h*^2^) was estimated as $h^{2}=\;\sigma_{g\;}^{2}/(\sigma_{g}^{2}+\;\sigma_{d}^{2}+\;\sigma_{\varepsilon }^{2})$. Where $\sigma_{g\;}^{2}$is additive genetic variance; $\sigma_{d}^{2}$ dominance variance; and, $\sigma_{\varepsilon }^{2}$ residual. Dominance to total variance ratio (*d*^2^) was estimated as $d^{2}=\;\sigma_{d\;}^{2}/(\sigma_{g}^{2}+\;\sigma_{d}^{2}+\;\sigma_{\varepsilon }^{2})$.

### Theoretical accuracy of breeding values

Theoretical accuracies of breeding values from ABLUP, ssGBLUP models using all the trees were estimated with the following expression.

$$r=\;\sqrt{\frac{1-\mathrm{PEV}}{\sigma_{a}^{2}(1+\;F_{i\;})}}$$

Where PEV is the prediction error variance from diagonal elements of the matrix from the mixed model equation (Gilmour *et al.* 1995), $F_{i\;}$ is the inbreeding coefficient of the tree I, $\sigma_{a}^{2}$ is the additive genetic variance from ABLUP and ssGBLUP models.

### Cross-validation tests

Cross-validation tests were performed to estimate the predictive ability and prediction accuracies of breeding values estimated with different models. Ten-fold cross-validation tests were performed to estimate the predictive abilities and prediction accuracies. In 10-fold cross-validation, all samples were divided randomly into ten folds. Samples from nine folds were used to develop the prediction model (training samples) which was then used to estimate the breeding values of the left-out fold (test samples) without the phenotypes. Predictive ability was estimated as the Pearson correlation between the predicted breeding values and the adjusted phenotypes of the test samples. Similarly, predictive accuracy was estimated as the Pearson correlation between the predicted breeding values from cross-validation with the estimated breeding values (EBVs) of test samples using marker and phenotype data of all the samples. This process is repeated ten times and the average of the 10 folds is used as the predictive ability and prediction accuracy of the model. When estimating prediction accuracies of a model, the predicted breeding values from cross-validation were correlated with the breeding values estimated with the data (marker and phenotype) of all the samples using that model.

Predictive ability and accuracies are estimated separately for MBVs/molecular genetic values (MGVs) from GBLUP models and EBVs/estimated genetic values (EGVs) from ABLUP models. Breeding values estimated with GBLUP-IBS are indicated as MBVs, MBVs with GBLUP-IBD are indicated as MBV_IBD, genetic values with GBLUP-AD are indicated as MGVs and genetic values estimated with the ABLUP-AD model are indicated as EGVs.

Predictive ability and accuracies of MBVs/MGVs are estimated by correlating MBVs/MGVs from cross-validation tests with the MBVs/MGVs estimated with all samples, respectively. Similarly, predictive ability and accuracies of EBVs/EGVs are estimated by correlating EBVs/EGVs from cross-validation tests with the EBVs/EGVs estimated with all samples, respectively.

Analysis of variance (ANOVA) was performed to test the differences in predictive ability and prediction accuracy of different models. A Tukey’s multiple comparison test was performed to identify the significant differences between different models. A significance level of α = 0.05 was used to test the differences between different models.

## Results

### Pedigree correction and reconstruction

GRM was used to identify pedigree errors and to reconstruct pedigree by identifying paternal parents of OP and polycross families. Sixty out of 868 trees showed pedigree errors. In total 133 individuals from 19 families were assigned paternal parents thus converting them into full-sib families. Without the pedigree reconstruction, these families would have been treated as half-sib families. Pedigree corrected progeny were used in all the downstream analyses.

### Genetic parameters of GBLUP and ABLUP models using 868 genotyped trees

The estimates of genetic parameters are lower with the increased utilization of genomic information (Table  2). Genetic parameters estimated with ABLUP are higher for all traits compared to GBLUP using the 868 genotyped trees. Between the two ABLUP models, the estimates of genetic parameters are higher for ABLUP without pedigree reconstruction compared to ABLUP-PR with pedigree reconstruction. The highest difference between ABLUP and GBLUP is observed for KPY. The heritability of KPY with ABLUP is 0.93 without the pedigree reconstruction, 0.70 with pedigree reconstruction while the heritability estimated with GBLUP is 0.30.

**Table 2:** Genetic parameters using 868 genotyped trees

Model	Trait	GBLUP-IBS		GBLUP-IBD		ABLUP-PR		ABLUP	
		Value	SE	Value	SE	Value	SE	Value	SE
	**DBH**								
**A**	Additive	0.17	0.04	0.17	0.04	0.41	0.09	0.45	0.10
	Residual	0.59	0.04	0.60	0.04	0.40	0.07	0.36	0.08
	*h* ^2^	0.23	0.05	0.22	0.05	0.50	0.10	0.56	0.11
	AIC	808		815		820		820	
**A + D**	Additive	0.11	0.04			0.41	0.10	0.45	0.12
	Dominance	0.12	0.04			0.00	0.17	0.00	0.23
	Residual	0.50	0.05			0.40	0.14	0.36	0.19
	*h* ^2^	0.16	0.05			0.50	0.12	0.56	0.13
	*d* ^2^	0.16	0.05			0.00	0.21	0.00	0.29
	AIC	787				820		820	
	**DEN**								
**A**	Additive	0.32	0.06	0.27	0.06	0.51	0.13	0.70	0.15
	Residual	0.78	0.06	0.84	0.05	0.61	0.10	0.42	0.12
	*h* ^2^	0.29	0.05	0.24	0.04	0.46	0.10	0.62	0.11
	AIC	809		816		812		796	
**A + D**	Additive	0.30	0.06			0.36	0.13	0.61	0.17
	Dominance	0.10	0.05			0.50	0.31	0.32	0.36
	Residual	0.69	0.07			0.25	0.26	0.19	0.29
	*h* ^2^	0.28	0.05			0.32	0.11	0.55	0.14
	*d* ^2^	0.09	0.05			0.45	0.27	0.29	0.32
	AIC	805				808		795	
	**KPY**								
**A**	Additive	0.31	0.06	0.29	0.05	0.81	0.15	1.09	0.18
	Residual	0.73	0.05	0.79	0.05	0.34	0.11	0.09	0.13
	*h* ^2^	0.30	0.05	0.27	0.04	0.70	0.11	0.93	0.11
	AIC	784		805		805		781	
**A + D**	Additive	0.29	0.06			0.81	0.17	1.09	0.22
	Dominance	0.03	0.04			0.00	0.24	0.00	0.34
	Residual	0.71	0.06			0.34	0.21	0.09	0.25
	*h* ^2^	0.28	0.05			0.70	0.12	0.93	0.15
	*d* ^2^	0.03	0.04			0.00	0.21	0.93	0.15
	AIC	783				805		781	

Dominance effects estimated with the GBLUP-AD model is significant for only DBH among the three traits (Table  2). For this trait, the magnitude of additive and dominance variances are equal with the GBLUP-AD model. The Akaike information criteria (AIC) of GBLUP-AD is lower than the GBLUP-A model for DBH indicating the better fit of the model with the dominance effects compared to the additive model.

Among the three traits, DEN had higher dominance effects with ABLUP-PR (pedigree reconstruction). Even though high dominance was observed with ABLUP (without the pedigree reconstruction), it was associated with high standard error (Table  2). The model fit with dominance effects has not changed compared to the additive only model for the ABLUP while it has improved marginally for the ABLUP-PR model. Dominance effects with GBLUP-AD are low for DEN (Table  2).

We also estimated the genetic parameters with GBLUP using the IBD GRM. Only additive effects are estimated with the IBD matrix. For DBH, the genetic parameters estimated with IBD are similar to the IBS matrix. For DEN and KPY the additive effects and heritability estimates with IBD are slightly lower than the IBS matrix (Table  2).

### Inbreeding depression

Inbreeding among the genotyped samples was estimated with the PLINK package. Inbreeding depression was estimated by linear regression between the inbreeding coefficients and the phenotype data. There is a significant negative relationship between inbreeding and DBH (Table  3). Similarly, there is a significant negative relationship between inbreeding and KPY. However, there is a nonsignificant positive relationship between inbreeding and DEN. These results suggest inbreeding depression for DBH and KPY. To test the linearity between inbreeding and the phenotypes, quadratic regression was performed. The quadratic coefficient was not significant for DBH indicating the linear relationship between inbreeding and DBH. However, for KPY there is a significant quadratic coefficient indicating nonlinearity of the relationship between inbreeding and KPY (Table  3). Significant quadratic regression between inbreeding and KPY indicates epistasis as a significant factor contributing to the inbreeding depression in this trait. To compare the best fit between linear and quadratic regression models for each trait, a partial *F*-test was performed. The partial *F*-test is not significant for DBH while it is significant for KPY (Table  3). This indicates that the linear regression is the best fit for DBH while the quadratic regression is the best fit for KPY.

**Table 3:** Coefficients of linear and quadratic regression between inbreeding and phenotypes of different traits; correlation between inbreeding coefficients and dominance deviation of different traits

Trait	Linear regression	Quadratic regression	Partial *F*-test
DBH	−2.09^***^	−1.76^NS^	3.34^NS^
KPY	−1.06^***^	−4.23^***^	13.26^***^
DEN	0.16^NS^	0.31^NS^	–
Correlation between dominance deviation and inbreeding			
DBH	−0.71		
KPY	−0.61		
DEN	0.23		

*** *P *<* *0.0001; NS, not significant.

To study the effect of inbreeding on dominance, dominance deviations from the GBLUP-AD model were correlated with the inbreeding. While DBH and KPY had a negative relationship with inbreeding, DEN had a positive relationship (Table  3). These results suggest trees with high DBH and KPY are less inbred.

### Predictive abilities and prediction accuracies of GBLUP and ABLUP models using 868 genotyped trees

**Figure 1:**
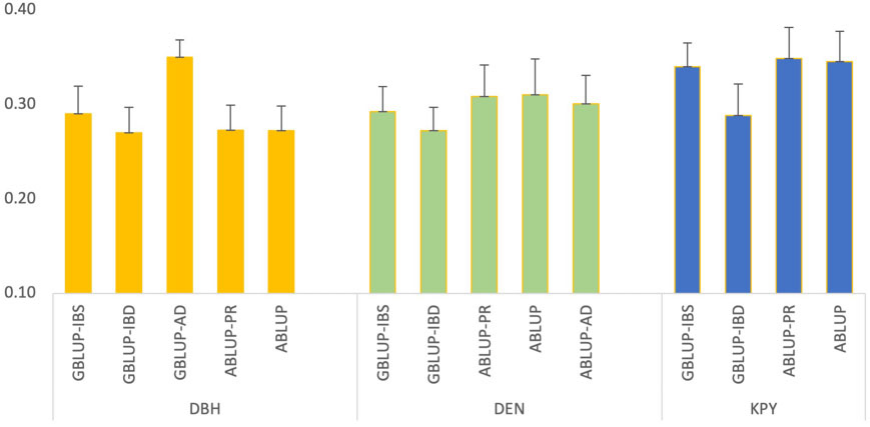
Predictive abilities of different models among 868 genotyped trees. ABLUP-AD for DEN is based on ABLUP-PR; ABLUP-AD for DBH, GBLUP-AD for DEN, GBLUP-AD, and ABLUP-AD for KPY are not shown as the dominance effects were close to zero for these traits.

Predictive ability indicates the ability of a model to predict the phenotypes while prediction accuracy indicates the ability of a model to predict the breeding values. For DBH, the predictive ability with the GBLUP-AD model is the highest compared to other models (Figure  1). Among the other four models, the predictive ability is similar. For DEN and KPY, predictive ability among different models is similar. While high dominance was observed for DEN with ABLUP models, it, however, did not result in higher predictive ability compared to the additive models. The predictive ability of GBLUP models with IBD and IBS matrices are the same for DBH while they are lower for GBLUP-IBD for DEN and KPY (Figure  1). However, none of these differences were significant with Tukey’s test at a significance level of α = 0.05.

**Figure 2:**
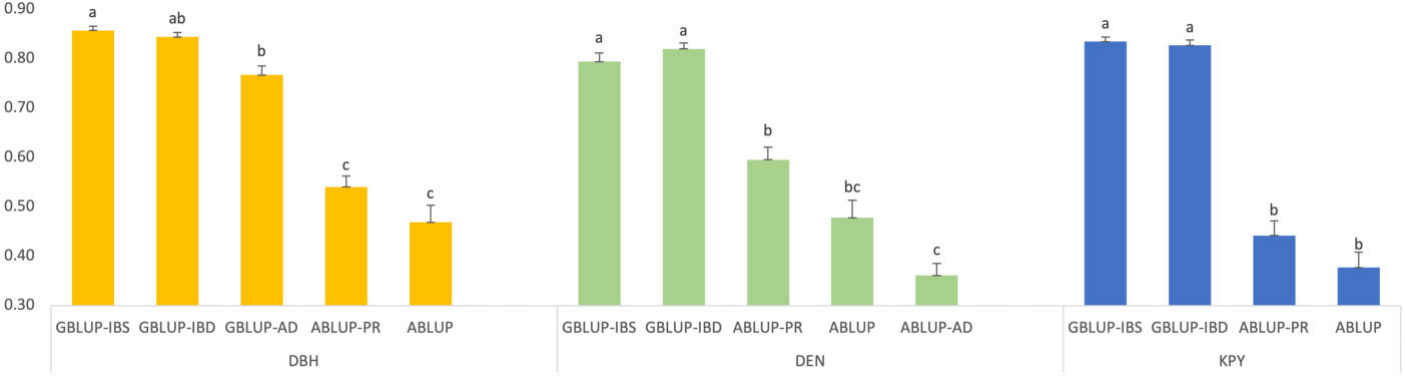
Predictive accuracies of different models among 868 genotyped trees. Accuracies with different letters indicate significant differences with Tukey’s test at a significance level of α = 0.05. ABLUP-AD for DEN is based on ABLUP-PR; ABLUP-AD for DBH, GBLUP-AD for DEN, GBLUP-AD, and ABLUP-AD for KPY are not shown as the dominance effects were close to zero for these traits.

Significant differences between GBLUP and ABLUP models were observed for prediction accuracies (Figure  2). However, between the two GBLUP models (with IBS and IBD matrices) there are no significant differences. For all traits, accuracies with pedigree reconstruction (ABLUP-PR) have improved over the ABLUP model. The significant dominance effect observed for DEN with the ABLUP-AD (with ABLUP-PR) model (Table  2) did not result in higher accuracy (Figure  2). The high genetic effects observed with the ABLUP-AD model (Table  2) did not result in high predictive ability or accuracy indicating the inflation of genetic parameters estimated with the ABLUP model.

### Accuracy of predicting breeding values (MBVs) and total genotypic values (MGVs) with additive only (A) and additive and dominance (A + D) models

**Figure 3:**
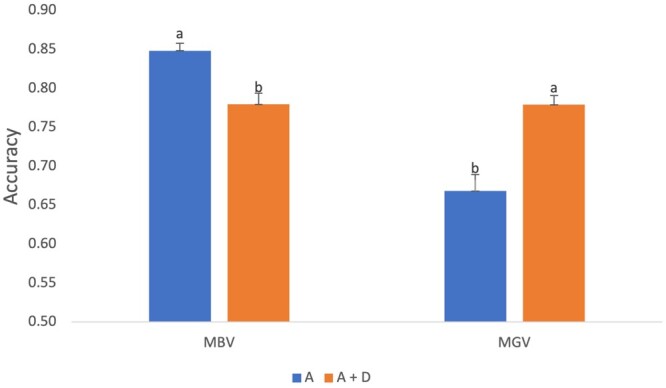
MBV and MGV accuracies with additive only (A) and additive and dominance (A + D) models. Accuracies with different letters indicate significant differences with Tukey’s test at a significance level of α = 0.05.

Next, we tested the accuracy of predicting MBVs and MGVs with an additive model (A) and additive and dominance model (A + D). Accuracy was estimated by correlating MBVs and MGVs estimated using all samples with MBVs/MGVs from cross-validation tests using additive (A) and additive and dominance (A + D) models. While there are significant differences between the models, the difference in the accuracy between the A and A + D models for MGVs is higher than MBVs (Figure  3). This indicates that the accuracy of predicting total genotypic values (MGVs) with the additive and dominance model (A + D) will be higher than the additive model while the accuracy of predicting breeding values (MBVs) will only be slightly better with the additive (A) model compared to the additive and dominance (A + D) model.

### Comparison of pairwise relationship coefficients of IBS and IBD matrices

The pair-wise relationship coefficients of the IBS matrix had higher dispersion than those of the IBD matrix (Table  4). For unrelated individuals, the lowest pair-wise relationship coefficients were zero with IBD while they were negative with the IBS matrix. The average of the diagonals was close to one with IBD while it is higher than one with the IBS matrix. Similarly, the standard deviations of diagonals and off-diagonals were lower with IBD compared to the IBS matrix.

**Table 4:** Pair-wise relationship coefficients of IBS and IBD matrices

	IBS	IBD
Avg	0.002	0.023
AvgDig	1.156	1.003
AvgOffDiag	0.001	0.023
stdDiag	0.098	0.025
stdOffDiag	0.074	0.045

Avg, average of all coefficients; AvgDig, average of diagonals; AvgOffDiag, average of off-diagonals; stdDiag, standard deviation of diagonals; stdOffDiag, standard deviation of off-diagonals.

### Genetic parameters with ssGBLUP and ABLUP models

In addition to GBLUP, we also used the ssGBLUP model to estimate genetic parameters with all 8821 genotyped and nongenotyped trees. Only DBH was used in the ssGBLUP model as data of DEN and KPY was not available for nongenotyped trees. Genetic parameters were estimated with the two relationship matrices (IBS and IBD) in ssGBLUP. In addition to ssGBLUP, a pedigree-based ABLUP model was also fitted. The ssGBLUP models had better model fit compared to ABLUP models as indicated by the lower AIC values of the ssGBLUP models. Between the two ssGBLUP models, ssGBLUP-IBS had a better model fit (Table  5). Higher heritability was observed with the ssGBLUP model using the IBS matrix compared to the other three models (Table  5). Heritability estimates of DBH with the ABLUP models using all trees (Table  5) were considerably lower than those with 868 genotyped trees (Table  2). Among the two ABLUP models, heritability estimates with the ABLUP-PR model are slightly higher than the ABLUP model.

**Table 5:** Genetic parameters estimated using 8821 genotyped and nongenotyped trees

	ssGBLUP-IBS		ssGBLUP-IBD		ABLUP-PR		ABLUP	
	Value	SE	Value	SE	Value	SE	Value	SE
Additive	0.57	0.06	0.28	0.04	0.26	0.04	0.23	0.04
Residual	0.95	0.05	1.17	0.04	1.19	0.04	1.21	0.04
*h* ^2^	0.38	0.04	0.19	0.03	0.18	0.03	0.16	0.03
AIC	7839		7843		7880		7891	

### Predictive abilities and prediction accuracies of ssGBLUP and ABLUP models using 8821 trees

**Figure 4:**
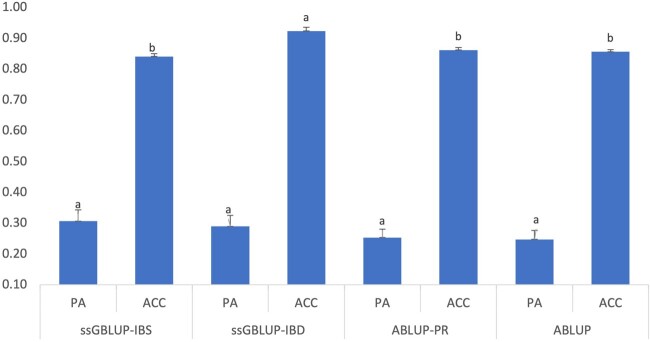
Predictive ability (PA) and prediction accuracies (ACC) among different models using DBH data of all 8821trees. Bars with different letters indicate significant differences between the models with Tukey’s test at a significance level of α = 0.05.

Cross-validation tests were performed to test the performance of ssGBLUP and ABLUP models using all 8821 trees. To reduce the influence of several nongenotyped trees and to compare the results from GBLUP analysis, tests were performed within the 868 genotyped trees. Prediction accuracies of ssGBLUP-IBD are significantly higher than the other three models (Figure  4). Prediction accuracies of ABLUP models with all 8821 trees (Figure  4) are considerably higher than those with 868 genotyped trees (Figure  2). The predictive ability of ssGBLUP models is slightly higher than the ABLUP models (Figure  4). Between the two ssGBLUP models, however, estimates of the predictive ability are similar (Figure  4).

### Theoretical accuracies among different models using 8821 trees

DBH data of all 8821 trees were used to compare the theoretical accuracies among the four models (ssGBLUP-IBS, ssGBLUP-IBD, ABLUP-PR, and ABLUP). Among all the four models, ssGBLUP-IBS had the highest accuracies among all three groups of trees (parents, genotyped, and nongenotyped—Table  6). Accuracies of ABLUP models are lower than the ssGBLUP models. Between the two ABLUP models, accuracies were higher (for genotyped and nongenotyped trees) with pedigree reconstruction (ABLUP-PR) compared to the model without pedigree reconstruction (ABLUP). Accuracy improved among all three groups (parents, genotyped, and nongenotyped trees) with the genomic models. Between the two genomic models, ssGBLUP-IBS had higher accuracies across all three groups compared to ssGBLUP-IBD. Theoretical accuracy of nongenotyped trees with ssGBLUP-IBS is the highest compared to the other three models (ssGBLUP-IBD, ABLUP-PR, and ABLUP) and among the three models, the accuracies are similar.

**Table 6:** Theoretical accuracies of different models using DBH data all 8821 trees

Group	ssGBLUP-IBS	ssGBLUP-IBD	ABLUP-PR	ABLUP
Parents	0.88	0.82	0.78	0.78
Genotyped	0.77	0.67	0.58	0.54
Nongenotyped	0.67	0.56	0.55	0.53

## Discussion

### Detection of significant dominance effects of DBH with preselected markers

In this study, we observed significant dominance effects for DBH with the GBLUP model using preselected markers. There is a concurrent improvement in the model fit with GBLUP-AD compared to the additive effects only model (Table  2). While not significant, there is a trend for higher predictive ability with the GBLUP-AD model compared to models with only the additive effects (Figure  1). In addition, the prediction accuracy of total genotypic values (MGVs) is higher with the GBLUP-AD model (Figure  3). These results are similar to those observed in *E. pellita* in our previous study (Thavamanikumar *et al.* 2020). However, the dominance effects observed in this study are in contrast to the results from other genomic studies in *E. nitens* (Klápště *et al.* 2017, 2018, 2020; Suontama *et al.* 2019). Klápště *et al.* (2017) did not observe dominance effects for DBH using sib-ship reconstructed pedigree. However, they did not use the GBLUP model with the GRM which captures realized relationships among the individuals within a family and between families. In other studies of *E. nitens* that implemented GBLUP, dominance effects were not estimated.

Preselected markers containing trait-relevant markers may have contributed to detecting dominance effects and higher accuracy of predicting phenotypes and total genotypic values of DBH in this study. In a previous study (Thavamanikumar *et al.* 2020), we observed significant dominance effects influencing growth traits in an OP population of *E. pellita* with the preselected markers. LD between a marker and the causal variant determines the additive and dominance effects observed for the marker (Aliloo *et al.* 2016). The additive variance observed at the marker depends upon the square of the correlation between the marker and the causal variant (*r*^2^), while the dominance variance observed at the marker depends on the *r*^4^ between the marker and causal variant. Thus, for detection of dominance effects markers that are in high LD with the causal variants are required (Wei *et al.* 2014). The accuracy of the models with the functional markers would persist across many generations (Habier *et al.* 2010).

Several studies have shown that the inclusion of dominance and trait-relevant markers improve prediction accuracies (Da *et al.* 2014; Beaulieu *et al.* 2014; Liu *et al.* 2019). Causal or trait-associated markers are important for the identification of dominance effects. Lenz *et al.* (2020) did not observe significant dominance effects using polycrosses and full-sib crosses in white spruce. While they suggested that the reason for the lack of dominance effects may be due to the small size of the population, another reason may be due to the un-selected markers used in their study. The accuracy of predicting total genetic values with causal markers is higher with a small number of causal markers than several random markers distributed across the genome (Da *et al.* 2014). Ramstein *et al.* (2020) have found that the inclusion of dominance and genic regions enriched with functional markers in prediction models improve prediction accuracies in maize hybrids. Low prediction accuracy was observed by excluding relevant markers and including irrelevant markers (Lippert *et al.* 2013). Klápště *et al.* (2020) have also shown higher accuracy of predicting traits in *E. nitens* and *Pinus radiata* using preselected markers. While these studies demonstrate the advantage of using trait-relevant markers in genomic predictions, identifying such markers is time-consuming and requires a large effort. We used high throughput methods and a discovery population consisting of trials from different sites and environments to detect trait-relevant markers. The marker panels used in this study consist of trait-relevant markers of different traits selected from thousands of markers by sequencing trait extreme samples from several trials.

### Relationship between inbreeding depression and dominance

DBH and KPY showed significant inbreeding depression (Table  3). Of these two traits, DBH had higher inbreeding depression. Other studies have also shown inbreeding depression of growth traits in *E. nitens* (Hardner and Tibbits 1998; Klápště *et al.* 2017). Inbreeding depression depends upon directional dominance and epistasis. Inbreeding depression or heterosis occurs due to directional dominance (Falconer and Mackay 1996). A trait should be influenced by dominance effects resulting in dominance variance to exhibit inbreeding depression (Howard *et al.* 2017; Doekes *et al.* 2021). Directional dominance is when the phenotypes of heterozygous individuals differ from homozygous individuals in a consistent direction. For fitness-related traits inbreeding depression is negative *i.e.*, fitness is reduced with the increased homozygosity (Yengo *et al.* 2017). Inbreeding depression of DBH may be due to directional dominance as indicated by the significant negative linear relationship between inbreeding and DBH (Table 3). Inbreeding depression in KPY may be influenced more by epistasis as indicated by the significant negative quadratic relationship between inbreeding and KPY (Table  3).

Dominance variance increases with increased inbreeding. Populations with high levels of inbreeding show a large proportion of the phenotype variation arising from dominance effects (Falconer and Mackay 1996). Misztal *et al.* (1997) identified higher inbreeding depression was associated with higher levels of dominance in cattle. There are two hypotheses explaining the inbreeding depression due to directional dominance. One is the partial dominance hypothesis in which deleterious recessive alleles which are masked under heterozygous condition are exposed with increased inbreeding leading to inbreeding depression. Another hypothesis is overdominance. Under this hypothesis the heterozygous condition itself is advantageous and this advantage decreases with increasing inbreeding leading to inbreeding depression (Crow and Kimura 1970; Charlesworth and Willis 2009).

It has been suggested to use inbreeding as a covariate in the genomic model to reduce the inflation of dominance variance (Vitezica *et al.* 2018). Inclusion of the inbreeding in the GBLUP-AD model as a fixed covariate reduced the dominance variance close to zero while not affecting the additive variance in this study (data not shown). Predictive ability with inbreeding in the GBLUP-AD model is lower (0.26—data not shown) than without the inbreeding (0.35—Figure  1). These results contrast with those of Xiang *et al.* (2016) who observed higher predictive ability with inbreeding in the genomic model compared to the model without the inbreeding. These results indicate that the dominance effects observed for DBH in this study are entirely due to inbreeding depression. The higher predictive ability of the GBLUP-AD model in this study may be mainly due to directional dominance effects of inbreeding depression. Including inbreeding in the genomic model absorbs the mean dominance across all loci leading to underestimation of the dominance. The dominance observed after accounting for inbreeding represents deviations of dominance effects of individual loci from mean dominance effects across all loci (Xiang *et al.* 2016; Doekes *et al.* 2020). This indicates that the dominance effects observed in this study are mainly due to mean dominance effects across the loci.

Correlation between dominance deviation and inbreeding provides a better estimate of the effect of dominance on inbreeding depression (Aliloo *et al.* 2016). There is a strong negative correlation between dominance deviations from the GBLUP-AD model and inbreeding coefficients for DBH and KPY in this study (Table  3). Similar results were also reported by Aliloo *et al.* (2016) for milk production traits in dairy cows. These results suggest that the trees with poor performance have gained less from dominance while trees with higher performance gained more from dominance. Therefore, selection based on dominance effects should identify the better performing trees.

### Bias of the genetic parameters estimated by ABLUP models

A high dominance ratio (*d*^2^) was observed for DEN with the ABLUP-PR model while the estimated dominance effect was low with the GBLUP-AD model (Table  2). However, the high dominance effect of the ABLUP-PR model did not result in higher predictive abilities (Figure  1) or higher prediction accuracies (Figure  2) using dominance effects in the prediction model. Klápště *et al.* (2017) have also observed high dominance effects for tangential air shrinkage of wood with sib-ship reconstruction in *E. nitens*. They did not see improvement in the model fit or precision of genetic parameters with the dominance relationship matrix in the model. Dominance effects with ABLUP models are not widely used as pedigree relationships are not sufficient. Large full-sib families are required for accurate estimates of the dominance. Moreover, prediction of dominance with pedigree information is cumbersome as it typically involves complex computations (Vitezica *et al.* 2013). Munoz and Sanchez (2014) indicated that estimation of genetic parameters with pedigree models is biased and genetic marker data should be used to partition additive and dominance effects.

Genetic parameters of DBH estimated with the ABLUP model using all 8821 (Table  5) trees are significantly lower than those estimated with 868 genotyped trees (Table  2). While the predictive ability has not improved, there is a large improvement in the prediction accuracy of DBH with ABLUP using all 8821 trees (Figure  4). This shows that the genetic parameters are overestimated with ABLUP using a small population with a few individuals per family compared to a large population with more individuals per family. Several studies have shown overestimation and low accuracy of the genetic parameters estimated with the ABLUP model (Muñoz *et al.* 2014; de Almeida Filho *et al.* 2019; Lenz *et al.* 2020). In tree breeding, most of the breeding populations are made up of shallow pedigrees with minimal connections between the families spanning only a few generations. Genetic parameters estimated with ABLUP models in such populations will be biased as only the higher-level relationships are utilized based on the pedigree information. On the other hand, the GRM used in the GBLUP captures the Mendelian segregation term among the individuals within a family as well as the hidden relationships between the families leading to higher accuracy of the genetic parameters and breeding values estimated with the GBLUP. The GBLUP, therefore, captures the contemporary as well as historical relationships leading to the high accuracy of the genetic parameters (Grattapaglia *et al.* 2018).

In several studies, the prediction accuracy of GBLUP is estimated by correlating MBVs with the EBVs of ABLUP. While this may be valid in animal breeding where deep pedigrees spanning several generations are used and the pedigree errors are minimal, this may not be useful in tree breeding with shallow pedigrees and several pedigree errors. Benchmarking the prediction accuracy of MBVs from GBLUP with less accurate EBVs will lead to biased estimates of the accuracy. This bias was highlighted by Klápště *et al.* (2018) in an OP population of *E. nitens* leading to a recommendation of benchmarking MBV accuracies of GBLUP with pedigree corrected EBVs of ABLUP. Even though this may result in better accuracies compared to those estimated without the pedigree correction, these accuracies are still biased. Pedigree reconstruction can only capture higher-level relationships such as half-sibs or full-sibs of a family, but within family Mendelian segregation term and lower-level relationships or the hidden relationships between families are not captured. Under these conditions, we propose benchmarking MBVs/EBVs from cross-validation with the MBVs estimated using all the samples used in the study as GRM captures all kinds of relationships including the hidden relationships. The MBVs estimated with all samples used in a study will therefore be closer to the true breeding values. In a recent study, Lenz *et al.* (2020) employed this approach to estimate the accuracies in white spruce.

### Theoretical accuracy of ssGBLUP and ABLUP models

Among all the four models using 8821 trees, ssGBLUP-IBS had the highest heritability (Table  5). While the estimates of heritability with ssGBLUP-IBS are higher than GBLUP-IBS, they are slightly lower for ssGBLUP-IBD compared to the GBLUP-IBD model (Tables  2 and 5). However, the predictive abilities and prediction accuracies of ssGBLUP models with 8821 trees (Figure  4) are similar to that of GBLUP models with 868 genotyped trees (Figure  1). While the ssGBLUP model did not result in higher predictive ability or higher prediction accuracy compared to GBLUP, there is a steady increase in the theoretical accuracy of ssGBLUP compared to the GBLUP models (data not shown). Among the four models (ssGBLUP-IBS, ssGBLUP-IBD, ABLUP-PR, and ABLUP), ssGBLUP-IBS had the highest theoretical accuracy among the three groups of the samples (parents, genotyped, and nongenotyped, Table  6). With ssGBLUP-IBS, accuracy has improved even in nongenotyped samples whereas the accuracy among the nongenotyped samples is similar between the other three models (ssGBLUP-IBD, ABLUP-PR, and ABLUP). The high heritability of ssGBLUP-IBS may reflect the improvement in the theoretical accuracy of DBH with ssGBLUP-IBS compared to the other three models. We observed similar results in an OP population of *E. pellita* (Thavamanikumar *et al.* 2020). These results are in contrast to the results of a study using an OP population in *E. nitens* (Klápště *et al.* 2018). In their study, no improvement in theoretical accuracy was observed among the parents and nongenotyped samples with ssGBLUP models compared to the ABLUP model. The lack of improvement in the accuracy was attributed to the pedigree errors and undetected hidden relationships among the nongenotyped samples. Higher theoretical accuracy of parents and nongenotyped samples with ssGBLUP-IBS in this study may be due to higher and more accurate estimates of heritability estimated with the preselected markers.

Theoretical accuracies reflect how much an individual’s breeding value improves with more data (Bijma 2012), while the prediction accuracies reflect the ability of the model to predict samples without the phenotype data. The similar prediction accuracy within the genotyped samples of ssGBLUP models compared to the GBLUP models may be due to the small proportion of genotyped samples compared to the total samples in ssGBLUP and the usage of a population with a shallow pedigree with minimal connectedness between the families. Another important factor could be pedigree errors. While pedigree errors in genotyped samples have been corrected, errors among nongenotyped samples may lead to biased estimates. In animal breeding where ssGBLUP is widely used pedigree errors are generally low. Moreover, genotype data from several thousand animals is available. Under these conditions ssGBLUP yields higher accuracy compared to GBLUP. Therefore, the accuracy of ssGBLUP in tree breeding may be improved by increasing the proportion of the genotyped samples and using breeding populations with deep pedigrees spanning several generations with several families. Ratcliffe *et al.* (2017) have shown increased improvement in the model fit, precision of genetic parameters, and breeding value accuracy with the increased proportion of the samples genotyped.

### Performance of ssGBLUP models with IBS and IBD matrices

In a recent study, Jurcic *et al.* (2021) have shown that implementation of a ssGBLUP with IBD relationship matrix resulted in better performance compared to a ssGBLUP model with IBS GRM in *E. dunnii*. Higher predictive ability was observed with IBD compared to the IBS matrix for DBH and stem straightness. They advocate the use of the IBD matrix in ssGBLUP analyses in OP tree evaluation. In this study, we compared the performance of the IBD and IBS matrices with GBLUP and ssGBLUP models. The predictive ability of IBS is higher than IBD with both GBLUP (Figure  1) and ssGBLUP (Figure  4) models even though these differences are not statistically significant. However, while the prediction accuracy is similar between the two matrices with GBLUP, there is a significant improvement in the prediction accuracy of the ssGBLUP-IBD model compared to the ssGBLUP-IBS model (Figure  4). Heritability and the theoretical accuracies estimated with ssGBLUP-IBS are higher than those with the ssGBLUP-IBD model. Moreover, the precision of the ssGBLUP-IBS model is better than the ssGBLUP-IBD model (Table  5). However, the dispersion of pair-wise relationship coefficients of ssGBLUP-IBD is lower than the ssGBLUP-IBS model (Table  4). While the high heritability of ssGBLUP-IBS, low dispersion of relationship coefficients of ssGBLUP-IBD and high prediction accuracy of ssGBLUP-IBD model are similar to the results of Jurcic *et al.* (2021), there is however no difference between the two models in predictive ability with both GBLUP (Figure  1) and ssGBLUP (Figure  4). The discrepancy in the performance of the models with IBD in the current study and the previous study may be due to the type of population and the type of markers used in the two studies. The breeding population used in this study consisted of parents and progeny from both CP and OP families while in the previous study an OP population was used. Parents of the progeny are also genotyped in this study. IBS based GS tracks information from many generations of pedigree leading to higher accuracy of IBS compared to IBD which is restricted to the documented pedigree (Luan *et al.* 2012). The relatively deep pedigree used in our study may be the reason for the better performance of the IBS matrix. Another issue may be LD or the marker-trait associations (functional variants) captured by the markers which leads to higher accuracy of IBS over IBD in GS models (Vela-Avitúa *et al.* 2015). The functional markers within the marker panel used in the current study may have contributed to improved performance of the IBS matrix over the IBD matrix in terms of predictive ability of GBLUP and ssGBLUP models; and the theoretical accuracy of the ssGBLUP model.

### Applications of dominance effects in tree breeding

Detection of significant dominance effects has practical implications in tree breeding. In tree species that can be vegetatively propagated, whole genotypic values which include both additive and dominance effects can be used in selection to transfer them to the next generation (de Almeida Filho *et al.* 2019). Dominance is also crucial for selecting complementary parents to exploit heterosis (de Almeida Filho *et al.* 2016). In species such as *E. nitens* where vegetative propagation is difficult, dominance can be used to select complementary parents for crossing. Optimal mate allocation involves selecting complementary parents to improve the genetic merit (which includes both additive and dominance effects) of the progeny. Simulation studies have shown substantial improvement in selection response with mate allocation techniques based on genomic models with dominance effects. However, the extra response is only observed in the first generation of mating and thereafter it is not further improved but maintained (Toro and Varona 2010). Mate allocation methods employing nonadditive effects will reduce the inbreeding in the progeny (Aliloo *et al.* 2017). In *E. nitens* controlled crossing between individual parents is difficult. Therefore, a polycross mating system is used to generate CP families with a pollen mix (polymix) collected from several parents. Even when full-sib crosses are possible, no advantage is found with full-sib crosses compared to polycrosses (Lenz *et al.* 2020). The total genotypic values (MGVs) of the parents may be used for generating CP families with improved progeny performance. In this study, among the top ten parents with the highest MGVs for DBH, one parent (maternal) has genotyped progeny. All the progeny derived from this parent have high MGVs (data not shown) indicating the inheritance of the dominance effects by the progeny. Controlled crossing among the top parents with the highest MGVs should, therefore, result in progeny with high MGVs and improved genetic performance.

## Conclusions

In this study, we observed significant dominance effects controlling DBH with preselected markers containing trait-relevant markers. Higher predictive abilities and higher total genotypic value accuracies were observed using prediction models with dominance effects. The performance of the models with IBS and IBD relationship matrices was similar within the genotyped samples using GBLUP. The prediction accuracy of IBD in ssGBLUP was higher than IBS. However, the theoretical accuracy of the ssGBLUP model with the IBS matrix is higher than the ssGBLUP model with the IBD matrix and ABLUP models. Improvement in theoretical accuracy was observed for nongenotyped samples and parents with the ssGBLUP-IBS model compared to other models. Significant inbreeding depression was observed for DBH and KPY. A linear relationship was observed between inbreeding and DBH while a nonlinear quadratic relationship was observed between inbreeding and KPY. This suggests that directional dominance contributes to the inbreeding depression of DBH while epistasis may be an important factor contributing to the inbreeding depression of KPY. The dominance effects observed for DBH in this study are mainly due to inbreeding depression. The significant dominance effect of DBH observed in this study may be used in mate allocation programs to select complementary parents to generate superior progeny.

## Data availability

The pedigree and trait data used in this study are in Supplementary Table S1. The marker genotype data used in this study are in Supplementary Table S2. Supplementary material is available at figshare: https://doi.org/10.25387/g3.15170883.
